# Persistence of serum and saliva antibody responses to SARS-CoV-2 spike antigens in COVID-19 patients

**DOI:** 10.1126/sciimmunol.abe5511

**Published:** 2020-10-08

**Authors:** Baweleta Isho, Kento T. Abe, Michelle Zuo, Alainna J. Jamal, Bhavisha Rathod, Jenny H. Wang, Zhijie Li, Gary Chao, Olga L. Rojas, Yeo Myong Bang, Annie Pu, Natasha Christie-Holmes, Christian Gervais, Derek Ceccarelli, Payman Samavarchi-Tehrani, Furkan Guvenc, Patrick Budylowski, Angel Li, Aimee Paterson, Feng Yun Yue, Lina M. Marin, Lauren Caldwell, Jeffrey L. Wrana, Karen Colwill, Frank Sicheri, Samira Mubareka, Scott D. Gray-Owen, Steven J. Drews, Walter L. Siqueira, Miriam Barrios-Rodiles, Mario Ostrowski, James M. Rini, Yves Durocher, Allison J. McGeer, Jennifer L. Gommerman, Anne-Claude Gingras

**Affiliations:** 1 Department of Immunology, University of Toronto, Toronto, ON, Canada.; 2 Lunenfeld-Tanenbaum Research Institute at Mount Sinai Hospital, Sinai Health System, Toronto, ON, Canada.; 3 Department of Molecular Genetics, University of Toronto, Toronto, ON, Canada.; 4 Institute of Health Policy, Management and Evaluation, University of Toronto, Toronto, ON, Canada.; 5 Department of Microbiology, at Mount Sinai Hospital, Sinai Health System, Toronto, ON, Canada.; 6 Combined Containment Level 3 Unit, University of Toronto, Toronto, ON, Canada.; 7 Mammalian Cell Expression, Human Health Therapeutics Research Centre, National Research Council Canada, Montréal, QC, Canada.; 8 Institute of Medical Science, University of Toronto, Toronto, ON, Canada.; 9 College of Dentistry, University of Saskatchewan, Saskatoon, SK, Canada.; 10 Department of Laboratory Medicine and Molecular Diagnostics, Division of Microbiology, Sunnybrook Health Sciences Centre; Biological Sciences, Sunnybrook Research Institute; and Division of Infectious Diseases, Sunnybrook Health Sciences Centre, Toronto, ON, Canada; Department of Laboratory Medicine and Pathology, University of Toronto, Toronto, ON, Canada.; 11 Canadian Blood Services, Edmonton, AB & Department of Laboratory Medicine and Pathology, University of Alberta, Edmonton, AB, Canada.; 12 St. Michael’s Hospital, Toronto, ON, Canada; Li Ka Shing Knowledge Institute.; 13 Department of Medicine, University of Toronto, Toronto, ON, Canada.; 14 Department of Biochemistry, University of Toronto, Toronto, ON, Canada.

## Abstract

While the antibody response to SARS-CoV-2 has been extensively studied in blood, relatively little is known about the antibody response in saliva and its relationship to systemic antibody levels. Here, we profiled by enzyme-linked immunosorbent assays (ELISAs) IgG, IgA and IgM responses to the SARS-CoV-2 spike protein (full length trimer) and its receptor-binding domain (RBD) in serum and saliva of acute and convalescent patients with laboratory-diagnosed COVID-19 ranging from 3–115 days post-symptom onset (PSO), compared to negative controls. Anti-SARS-CoV-2 antibody responses were readily detected in serum and saliva, with peak IgG levels attained by 16–30 days PSO. Longitudinal analysis revealed that anti-SARS-CoV-2 IgA and IgM antibodies rapidly decayed, while IgG antibodies remained relatively stable up to 105 days PSO in both biofluids. Lastly, IgG, IgM and to a lesser extent IgA responses to spike and RBD in the serum positively correlated with matched saliva samples. This study confirms that serum and saliva IgG antibodies to SARS-CoV-2 are maintained in the majority of COVID-19 patients for at least 3 months PSO. IgG responses in saliva may serve as a surrogate measure of systemic immunity to SARS-CoV-2 based on their correlation with serum IgG responses.

## Teaser

Saliva is an alternative biofluid to serum for detecting and monitoring IgG to SARS-CoV-2 spike and RBD antigens in COVID-19.

## INTRODUCTION

Antibodies play an important role in neutralizing virus and provide protection to the host against viral re-infection. The antibody response to SARS-CoV-2 infection has been extensively studied in the blood (serum, plasma) of COVID-19 patients in order to gain insights into the host immune response. Antibody levels to the spike protein are particularly important since this large trimeric glycoprotein harbors the receptor-binding domain (RBD). The RBD facilitates SARS-CoV-2 access to human cells by binding to its counter receptor angiotensin-converting enzyme 2 (ACE-2) (*1*), and neutralizing antibodies have been shown to target the RBD (*2*). Most studies agree that the IgG antibodies to SARS-CoV-2 spike and RBD antigens are detected in the blood of greater than 90% of subjects by 10–11 days post-symptom onset (PSO) (*3*–*7*). However, whether levels of IgG specific for SARS-CoV-2 antigen persist (*8*–*13*), or alternatively decay (*14*), remains a debated issue. Examination of different biofluids from multiple cohorts, and attention to the antigens tested, is required to resolve this extremely important issue that has high relevance to vaccine design.

Another gap in our knowledge is that we know very little about the local antibody response at the site of infection. SARS-CoV-2 enters the naso- and oro-pharyngeal tracts where it subsequently replicates (*15*). For this reason, nasopharyngeal and throat swabs are used to test for virus using reverse transcriptase quantitative PCR (RT-qPCR) to detect viral RNA. However, saliva has also been shown to be an effective biofluid for testing for the presence of SARS-CoV-2 mRNA (*16*–*19*). This makes sense given that pharyngeal SARS-CoV-2 shedding precedes viral replication in the lungs (*15*), and, like cytomegalovirus (*20*, *21*), the salivary glands themselves can be a reservoir for the virus (*22*). Yet in spite of the oral cavity being a site for viral replication, few studies have examined anti-SARS-CoV-2 antibodies in this compartment.

In this study, we examined the anti-SARS-CoV-2 antibody response over a 115-day period in the serum and saliva from n=439 (serum) and n=128 (saliva) patients with COVID-19, compared to controls. Antigen-specific IgG in both biofluids was maximally detected by 16–30 days PSO and did not drastically decline in relative level as late as 105-115 days PSO. In contrast, antigen-specific IgM and IgA were rapidly induced but subsequently declined in both serum and saliva. In serum, neutralizing antibodies reached their maximum by 31–45 days PSO and slowly declined up to 105 days, with a more pronounced drop in the last blood draw (105–115 days PSO) Importantly, IgG and IgM levels against both antigens were strongly correlated across paired serum and saliva samples (n=71), indicating that saliva can be used for monitoring the immune response to SARS-CoV-2 infection. Taken together, the systemic and mucosal IgG response to SARS-CoV-2 is sustained over a 3-month period, while the IgM and IgA response occurs early and is transient.

## RESULTS

### A chemiluminescent fully automated method for detecting antibodies to SARS-CoV-2 antigens in the serum of acute and convalescent patients

To study the antibody response to SARS-CoV-2, we initially focused on antibodies (IgM, IgG, IgA) to the spike homotrimer and the RBD, since neutralizing antibodies are directed to the spike protein (*23*). Enzyme-linked immunosorbent assays (ELISAs) for the detection in serum (or plasma) of anti-spike trimer and anti-spike RBD antibodies were built as in (*3*, *24*) as 96-well colorimetric assays, and implemented as automated 384-well chemiluminescence assays. For all serum-based assays, blank-subtracted colorimetric or chemiluminescent values were normalized to a pool of convalescent sera added to each assay plate, and expressed as ratios to this pool of positive samples (ratio-converted ELISA reads; see Methods). Receiver-Operating Characteristic (ROC) curves were generated on cohorts of true negatives (banked samples collected pre-COVID, n=339 for manual and automated assays) and positives (convalescent patients with confirmed PCR diagnostic, n=402 for manual and automated assays, see Table 1). For manual and automated IgG assays, sensitivities of 95.6% and 95.5% for spike and 93.8% and 91.3% for RBD, respectively, at a false positive rate of ≤1%, were obtained in these cohorts (Figure S1A-B, and Table S1 for ROC statistics). The Areas Under the Curves (AUCs) were ≥0.97 in all cases, indicating excellent assay performance. Automated assays for the detection of IgA and IgM were also developed (Figure S1C-D), though the sensitivity/specificity characteristics were lower than those of the IgG assays at least in part because, as is described below, these antibody responses wane more rapidly. The results for the automated and manual IgG assays were well correlated (Figure S1E-F).

**Table 1:** Cohorts of patients and negative controls.

	**SALIVA**						**BLOOD**					
		No. patients	No. samples	Median Age	Sex			No. patients	No. samples	Median Age	Sex	
					No. M	No. F					No. M	No. F
**All samples**		247	263	-	141	106	**All samples**	739	796	-	379	360
Patients with COVID-19	Cohort 1	47	54	61	28	19	Patients with COVID-19	439	496	58	229	210
	Cohort 2	81	90	58	48	33						
Pre-COVID Negative Controls	Cohort 1	0	0	0	0	0	Pre-COVID Negative Controls	300	300	54.5	150	150
	Cohort 2	27	27	43	12	15						
Unexposed Negative Controls Collected in 2020	Cohort 1	42	42	60	24	18						
	Cohort 2	50	50	58	29	21						
*Matched saliva-serum samples*		71	71	58	33	38						

**Fig. 1:**
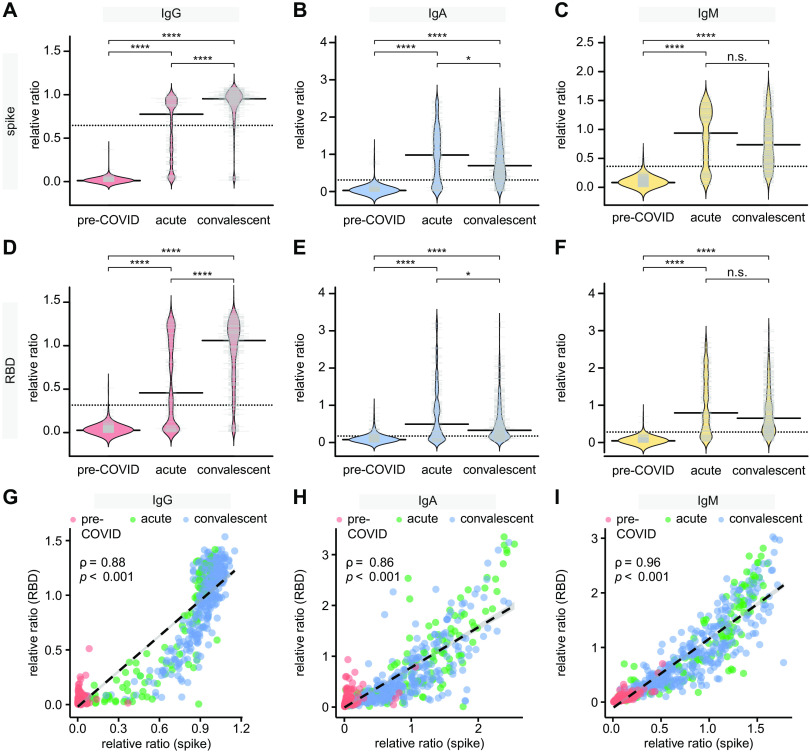
Cross-sectional analysis of IgG and IgA responses to the spike and RBD antigens of SARS-CoV2 in serum. (**A-F**) Indicated immunoglobulins to spike and RBD were profiled by ELISA in cohorts of pre-COVID samples (n=300), hospitalized patients with acute COVID infection (n=132) and convalescent patients (n=364). All data, expressed as ratio-converted ELISA reads to a pool of convalescent samples (relative ratio), were plotted using bean plots. Solid bars denote the median and dotted line represents the median across all samples used in the plot. (**G-I**) levels of IgG (G), IgA (H) and IgM (I) to the RBD (y-axis) and spike (x-axis) antigens for the indicated patient groups. Spearman correlation coefficient is indicated. Mann-Whitney U test for significance was performed. n.s = not significant, *= p ≤ 0.05, **** = p < 0.0001.

These automated ELISA assays were used to profile cohorts of confirmed acute and convalescent sera from COVID-19 patients collected as part of COVID-19 surveillance by the Toronto Invasive Bacterial Diseases Network (Table 1). As expected, based on the ROC analysis the convalescent and pre-COVID controls had very different ratio distributions for both antigens (Fig. 1A, D). On the other hand, serum collected from patients less than 21 days PSO (acute serum, n=132) had bimodal distributions in their IgG responses for both antigens (with an overall lower mean), suggesting that antibody concentrations were increasing over time. To compare the relationship between RBD and spike trimer IgG levels, we plotted their values against each other. While there was an overall high correlation between the antigens (Fig. 1G), we noted many more acute specimens with high spike-trimer and low RBD responses than vice versa, consistent with the fact that RBD is included within the spike trimer antigen. The concentration of IgA and IgM in convalescent serum was also clearly higher than that of the pre-COVID samples, but the acute cases had a higher median than the convalescents (Fig. 1B & E, C & F). The IgA and IgM levels to RBD and spike were also well correlated (Fig. 1H-I).

**Fig. 2:**
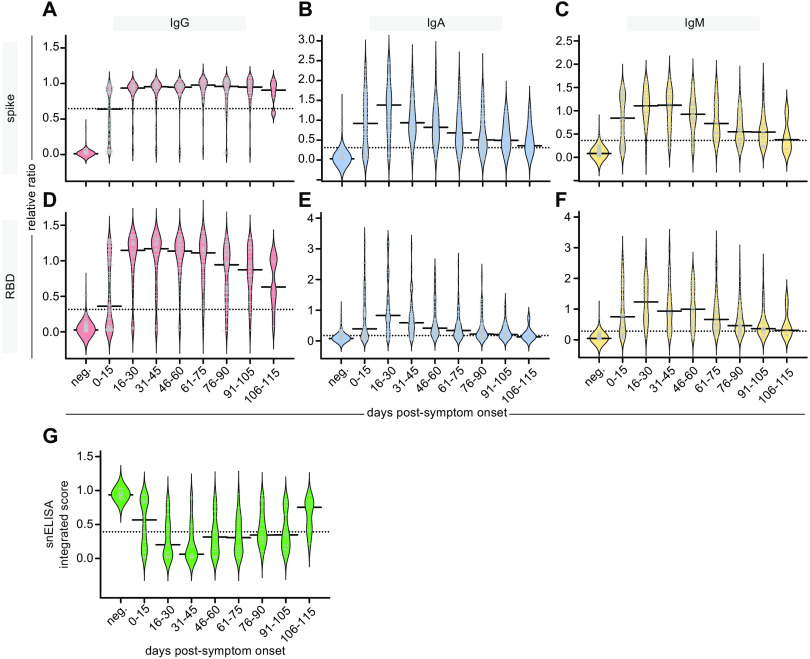
**Persistence of antibodies in the serum of affected individuals. (A-F**) Binned ratio-converted ELISA reads (relative ratios to a pool of positive controls) of spike (**A-C**) and RBD (**D-F**) to the indicated antibodies, displayed as bean plots. (**G**) The results of the surrogate neutralization ELISA are also shown, expressed as an integrated score tabulating the area under the curve across the first two points of the dilution series (see Methods). Days PSO are binned in 15-day increments and are compared to pre-COVID samples (neg). Solid bars denote the median and dotted line represents the median across all samples used in the plot. For A–F, the number of samples per bin was: neg=300; 0–15=115; 16–30=41; 31–45=50; 46–60=71; 61–75=62; 76–90=100; 91–105=9. For G, all bins were n=20, with the exception of neg.=19 and 106–115=9 (all available samples).

**Fig. 3:**
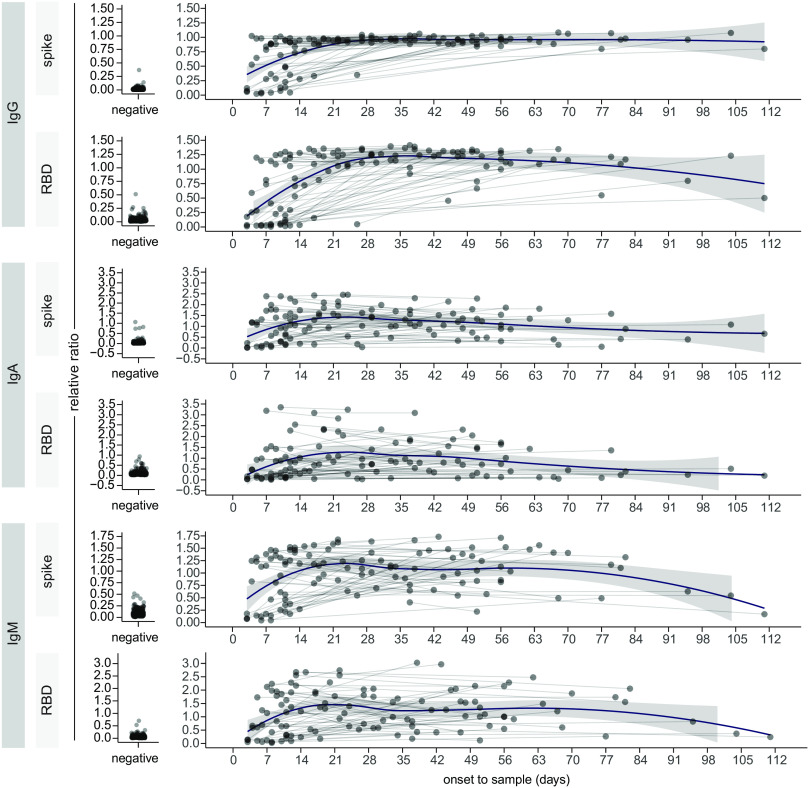
A longitudinal analysis of IgG and IgA responses to the spike and RBD antigens of SARS-CoV2 in serum. Analysis of the changes in the indicated Ig-antigen levels in patients profiled twice, in comparisons to the relative levels in pre-COVID negative controls (left). Dots represent individual serum samples collected at the indicated times, and the samples from the same patients are connected by the lines. A non-parametric loess function is shown as the blue line, with the grey shade representing the 95% confidence interval.

The bimodal distribution of the IgG responses in the acute serum (Fig. 1A, D), along with the different patterns of response for IgG versus IgA/IgM in acute and convalescent specimens (Fig. 1B & E, C & F), prompted us to plot the antibody levels against days PSO. Spearman rank correlation analysis revealed an overall increase in the IgG response versus a decrease in the IgA and IgM response to both antigens over time, and the IgG response in particular did not appear to be linear (compare panels A-B to C-D and E-F in Figure S2; IgG results were reproduced in the analysis of the manual IgG assays, shown in panels G-H). To look at this response more closely, specimens were binned by days PSO (15-day intervals), and the levels of the different immunoglobulins were plotted (the pre-COVID negative control samples were plotted alongside for comparison; Fig. 2). As was reported in other studies (*3*, *4*, *7*), the IgG levels peaked in the 16–30 days bin, and the levels of IgG against spike trimer appeared relatively sustained over 115 days (Fig. 2A). IgG levels against RBD showed a ~25.3% decrease by day 105, and ~46.0% by day 115 (Fig. 2D). IgA and IgM levels to both antigens were by contrast much less sustained: after reaching a maximum in the 16–30 days bin, there was a clear and continuous decline throughout the time series such that by 115 days, the anti-spike and anti-RBD IgA levels were ~74.1% and ~84.2% of their respective maximal levels, while IgM levels were ~66.2% and ~75.1%, respectively (Fig. 2B, E & C, F). Multivariable analyses adjusting for severity of illness, sex, and patient age, did not change conclusions about the aforementioned relationships between time PSO and anti-RBD IgM, anti-spike IgM, anti-RBD IgA, anti-spike IgA, and anti-RBD IgG; however, the modest decline in anti-spike IgG after day 35 was statistically significant (data not shown). The relative stability of the IgG anti-spike trimer levels, partial decrease in the anti-RBD IgG and anti-spike IgA levels, and a near complete loss in the anti-RBD IgM and IgA levels over time results were also detected in pairs of serum samples from hospitalized patients (n=57), collected at admission and 3–12 weeks later, using a nonparametric loess analysis (Fig. 3 as in (*25*)).

Although our focus was on the spike protein, we also examined the antibody response to nucleocapsid (a.k.a. nucleoprotein, NP), since this is the antigen targeted by multiple commercial assays. We developed an assay using bacterially-expressed NP (Figure S3A–C). When we examined the levels of anti-NP antibodies binned for time PSO, we found that their patterns closely resembled those for anti-spike and anti-RBD IgG and IgA/IgM responses, namely a relative stability in the IgG and more rapid decline in IgA/IgM levels in both the binned time series and the longitudinal series (Figure S3D-F).

To evaluate the neutralization potential of these antibodies, we used our recently established protein-based surrogate neutralization ELISA (snELISA) approach (Fig. 2G; (*24*)). Briefly, the snELISA measures the ability of antibodies (in serum in our case) to prevent the association of soluble biotinylated ACE2 to immobilized RBD: a higher signal (snELISA integrated score) in this assay indicates low neutralization. Using the binned time series as above, we report that the neutralization reaches its maximum in the 31–45 day PSO bin, and decreases to an intermediate median plateau in the 46–105 day PSO bins before more drastically dropping in the 106–115 day PSO samples (we note, however, that fewer samples are in this time bin (n=9) compared to the other bins (n=20); Fig. 2G).

In summary, in a large cross-sectional survey, IgG, but not IgA or IgM levels persisted for at least 3 months PSO for all antigens measured, with the levels of antibodies to the spike trimer being more stable over time than those to the RBD and NP. Neutralizing antibodies levels mirrored these antibody levels, though the drop observed in the last bin (105–115 days PSO), which was not as powered as the other bins, will need to be investigated more closely.

### Antibodies to SARS-CoV-2 antigens are detected in the saliva of COVID-19 patients

While our serum-based assays are scalable and robust, saliva represents a relatively unexplored biofluid for detecting antibodies to SARS-CoV-2 antigens with many practical benefits, including being noninvasive and the capacity for self-collection at home. The disadvantage of saliva as a biofluid is its very low concentration of antibodies (*26*), making it necessary to optimize the sensitivity of detection. We explored various assay designs and found that capturing biotinylated spike and RBD antigens on streptavidin-coated plates (rather than adsorbing non-biotinylated proteins directly on the ELISA plates) was required to obtain reliable signal-to-noise ratios. This method also required that the saliva be pre-adsorbed to remove any streptavidin-binding protein. While heat (65°C for 30 min) prevented detection of antibodies in the saliva, treatment of saliva samples with Triton X-100 was compatible with our assay (Figure S4) and resulted in viral inactivation (Table S2). Bolstered by these findings, we first performed a pilot experiment, using expectorated saliva samples acquired during the early phase of the pandemic, measuring antibody levels to SARS-CoV-2 antigens in n=54 COVID-19 patients (cohort 1), comparing to unexposed negative controls collected locally (n=42). Since these samples were diluted to varying degrees, we normalized values to total IgG/IgA (depending on the isotype assay) or to albumin levels as done before by others (*27*). The mean, standard deviation and concentration range of total IgA and IgG from the COVID-19 patients were 60.2 ± 99.2 μg/ml (4.6 μg/ml – 656.9 μg/ml) and 25.5 ± 47.7 μg/ml (2.5 μg/ml – 275.1 μg/ml), respectively. The mean, standard deviation and concentration range of total IgA and IgG from the unexposed negative controls were 89.3 ± 72.7 μg/ml (7.0 μg/ml – 452.9 μg/ml) and 7.0 ± 7.8 μg/ml (2.4 μg/ml – 48.8 μg/ml), respectively. The mean, standard deviation and concentration range of albumin from the COVID-19 patients and unexposed negative controls were 9.6 ± 8.1 μg/ml (1.3 μg/ml – 32.6 μg/ml) and 9.3 ± 9.4 μg/ml (1.2 μg/ml – 45.8 μg/ml), respectively. Saliva samples from COVID-19 patients displayed a significantly higher level of IgG and IgA levels to spike and RBD compared to negative controls when normalized with either method (Figure S5).

Following this pilot experiment, we proceeded with further saliva collections using Salivettes® to standardize our collection method without using a diluent (cohort 2) in order to measure IgG, IgA and IgM levels to both spike and RBD antigens. In cohort 2, we obtained n=90 samples from 80 patients ranging in time PSO from day 3–104. These were compared to 50 unexposed negative controls for cohort 2, of which 42 were also negative controls for cohort 1. To these negative controls, we also added pre-COVID era saliva samples as an additional comparator (n=27). Our antigen assays had a working volume of 50 μl in each well, and in these assays, we measured anti-spike and anti-RBD antibodies in the samples at three dilutions: 1/5, 1/10 and 1/20. In every experimental plate, we ran a positive control (pooled saliva from several COVID-19 patients) and negative control (pooled saliva from unexposed subjects) also plated at 1/5, 1/10 and 1/20. We measured the area under the curve of every sample and performed a normalization to the internal plate controls as shown in Figure S6. We reported the normalized values as a percentage of the positive control (denoted as “integrated score”). While we did not normalize to total Ig levels in cohort 2, we still measured Ig levels in the saliva of COVID-19 patients and negative controls. The working volume of these experiments was 50 μl and several different dilution series were run for each sample, depending on the antibody isotype being measured, to best determine total IgA/M/G concentrations.

**Fig. 4:**
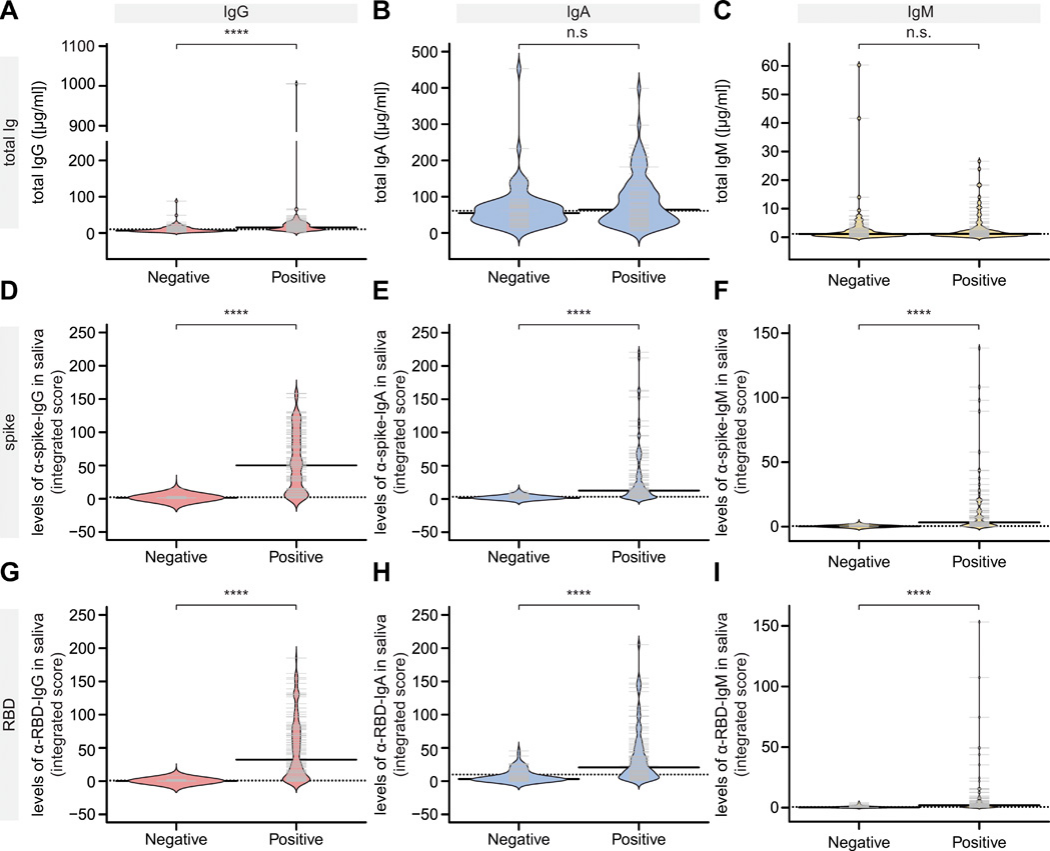
Cross-sectional analysis of antibody responses to the spike and RBD antigens of SARS-CoV-2 in saliva. Saliva specimens from the cohort of COVID-19 patients were tested for the presence of IgG, IgA and IgM antibodies to SARS-CoV-2 spike and RBD antigens (Positive), comparing with a mixture of unexposed asymptomatic controls collected locally and pre-COVID era controls (Negative). In these cohort 2 samples collected in Salivettes® we had sufficient material to perform several dilutions and to generate an integrated score for each subject (see Methods). Because the saliva was not diluted during collection, we were able to derive the concentration of antibodies in both negative controls and COVID-19 patients. (**A-C**) Total IgG, IgA and IgM levels in the saliva. (**D-I**) Saliva data for negative controls versus COVID-19 patients. Solid bars denote the median and dotted line represents the median across all samples used in the plot. Mann-Whitney U test for significance was performed. **** = p < 0.0001, n.s. = not significant.

Total IgG levels, but not IgA or IgM levels, were found to be higher in COVID-19 patients compared to controls (Fig. 4A-C). Moreover, cohort 2 exhibited statistically significant differences between the relative levels of IgG, IgA and IgM antibodies specific to spike and RBD antigens compared to saliva from negative controls (Fig. 4D-I). The sensitivity of the saliva assays for IgG antibodies to spike and RBD (at a false discovery rate <2%) were 89% and 85%, respectively, while the sensitivity of the assays for IgA antibodies to spike and RBD were 51% and 30%, respectively, and the sensitivity of the assays for IgM antibodies to spike and RBD were 57% and 33%, respectively. (Figure S7 and Table S3). The lower sensitivity of the IgA assays is attributed in part to the higher levels of anti-spike and anti-RBD IgA levels in the negative controls (see Discussion).

**Fig. 5:**
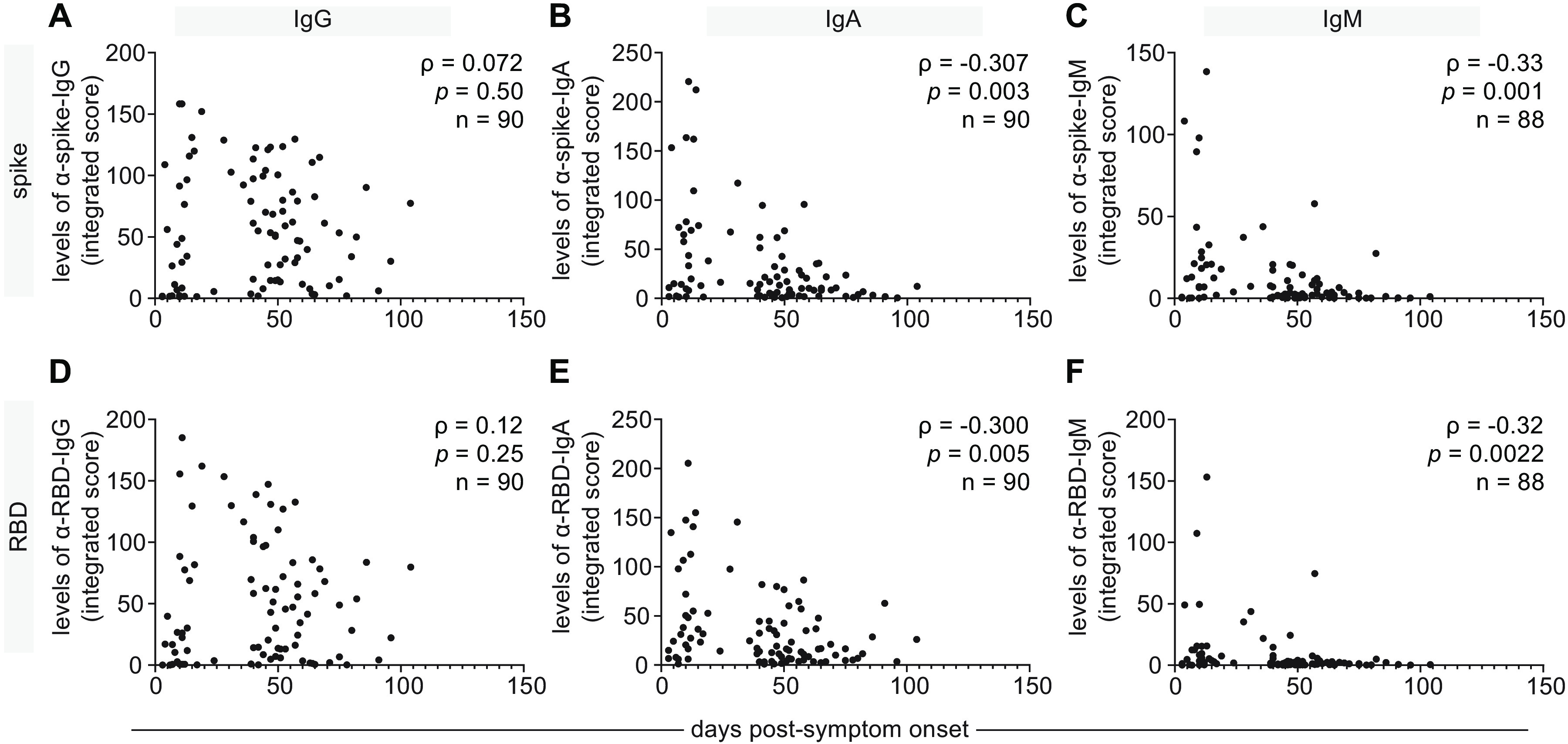
A cross-sectional analysis of antibody responses to the spike and RBD antigens of SARS-CoV-2 in saliva correlated with time PSO. A second cohort of COVID-19 patients (n=90) was tested for the presence of IgG and IgA antibodies to SARS-CoV-2 spike and RBD antigens in the saliva, comparing with a mixture of unexposed negative controls collected locally and pre-COVID era negative controls. (**A-F**) Saliva data for all 6 antigen-specific ELISA readouts plotted as time PSO. Spearman correlation coefficients (ρ) and p-value are indicated. In multivariable analysis adjusted for age, sex and severity of illness, there was a significant decline in anti-RBD and anti-spike IgA, but not significant change in the level of anti-RBD or anti-spike IgG.

Next, we examined the levels of anti-spike and anti-RBD antibodies in our cross-sectional cohort over time PSO. Similar to the serum data, IgG levels in saliva to the spike and RBD antigens remained stable throughout the 3-month collection period. In contrast, significant decreases were observed for IgA levels to spike and RBD (ρ=-0.307 and ρ=-0.300, respectively), and similar results were observed for IgM levels to spike and RBD (ρ=-0.33 and ρ=-0.32, respectively). By day 100, anti-spike and anti-RBD IgA and IgM levels were barely detectable (Fig. 5). In summary, infection with SARS-CoV-2 results in detectable IgG, IgA and IgM responses in saliva against the spike and RBD antigens, with only the IgG response persisting beyond day 60.

### Antibody levels to SARS-CoV-2 antigens in the serum correlate with those in the saliva

**Fig. 6:**
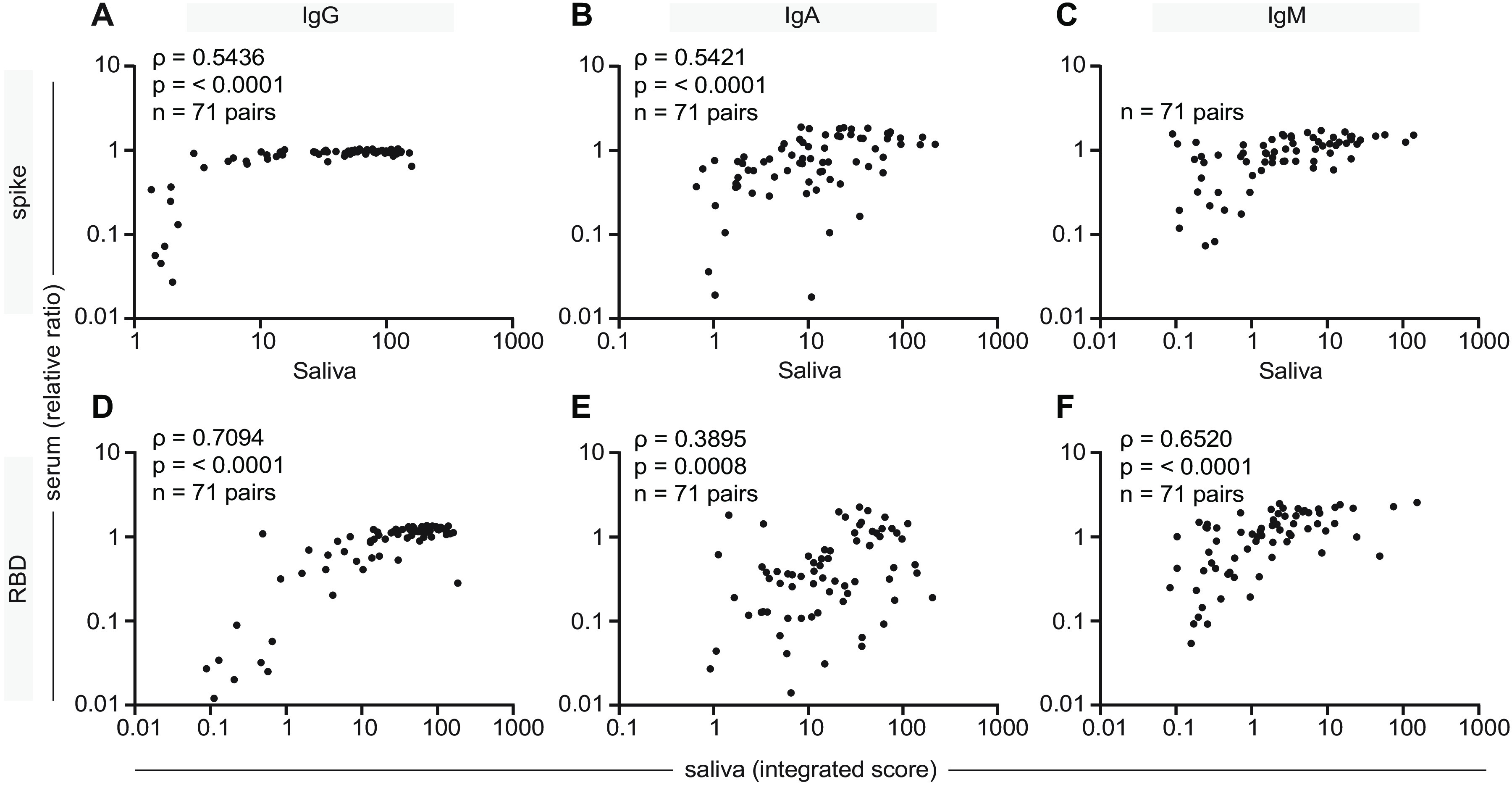
**Correlation of IgG, IgA and IgM responses to the spike and RBD antigens in serum and saliva. (A-F)** A subset of serum and saliva sample pairs (n=71) collected from the same patient within 4 days were analyzed for correlations in levels of anti-spike and anti-RBD IgG, IgA and IgM antibodies. For serum, data are presented as ratio-normalized ELISA reads, while the saliva results are expressed as an integrated score, as in previous figures. The data are presented on a logarithmic scale. Spearman correlation coefficient (ρ) and p-value are indicated.

As mentioned, saliva has many advantages for biofluid collection over serum. To assess whether saliva might be reliably used in a diagnostic test, we determined whether the antibody levels to spike and RBD in the saliva correlated with those measured in the serum. Of the COVID-19 patients analyzed, n=71 had paired saliva and serum samples taken at similar timepoints (i.e., within 4 days). We observed a significant positive correlation between saliva and serum for each antigen-antibody combination (Fig. 6; values are plotted on log scales; see legend for details). Correlations for anti-RBD and anti-spike IgG (ρ=0.71, ρ=0.54), and anti-RBD and anti-spike IgM (ρ=0.65, ρ=0.58) were stronger than those for the levels of serum and saliva anti-RBD and anti-spike IgA (ρ=0.39 and ρ=0.54 respectively). Therefore, at least for anti-spike IgM and anti-RBD IgG measurements, saliva may represent a good alternative for antibody testing.

## DISCUSSION

Antibodies are key components in the arsenal of protective immunity against novel viral infections such as SARS-CoV-2. Understanding their durability and their system compartmentalization across a diverse population are critical pieces of data informing our ability to monitor seroprevalence in communities, to select plasma donors for treatment, and to design vaccines against COVID-19. We examined the stability of antibody levels over the first three months after infection in both the serum and the saliva. We observed no drastic decline in levels of anti-spike, anti-RBD or anti-NP IgG levels over a 3-month period. The same was true for the antigen-specific measurements in saliva (anti-spike and anti-RBD IgG). On the other hand, similar to other findings (*28*, *29*), IgA and IgM responses to SARS-CoV-2 antigens were found to decline in both serum and saliva. In summary, our data show that a durable IgG response against SARS-CoV-2 antigens is generated in both the saliva and serum in most patients with COVID-19. Of the three isotypes measured, the IgA response correlates the least between serum and saliva, particularly for the RBD antigen. This may suggest some compartmentalization of the IgA response in the oral cavity versus the periphery.

Given the presence of SARS-CoV-2 RNA in saliva, it is reasonable to hypothesize that, like other viruses such as rubella (*26*), 229E alpha-coronavirus (*30*), and MERS beta-coronavirus (*31*), the mucosae and draining lymph nodes of the oro- and nasopharyngeal tracts serve as a site for initiation of an immune response to SARS-CoV-2. If so, then plasma cells (PC) that produce antibodies to SARS-CoV-2 will migrate back to the oro- and nasopharyngeal mucosae and produce antibodies that should be detectable in the saliva, a fluid that already has high levels of IgA (*32*). With time, this response will be detected in the systemic circulation, possibly due to migration of PC into new niches as we have previously described in mice (*33*). Indeed, we and two other groups have observed SARS-CoV-2 specific antibodies in saliva (*34*, *35*). There are some variations between study protocols that are important to consider: Randad *et al*. applied a brush on the gum line as a means to capture IgG from the blood, heat inactivated this material, and performed multiplex antibody immunoassays using Luminex technology to detect antigen-specific antibody levels (*35*). In contrast, our strategy was to collect saliva in a manner that best approximates the immune response that takes place in the local mucosa. In this way, our study more resembles that of Faustini *et al*., who used ELISA technology on whole saliva, amplifying the signal with an additional antibody step (*34*). Although Faustini *et al*. employed saliva dilutions in the same range as what we used (1:5 to 1:20), the degree of correlation between the serum and saliva for each antibody/antigen ELISA pair was less obvious in that study than in ours (*34*). Whether these discrepancies are methodological (i.e., detection of specific versus total Igs) and/or relate to the higher number of asymptomatic subjects in the Faustini *et al*. study remains to be determined.

While the specificity of the saliva assays was very good for anti-spike and anti-RBD IgG responses based on ROC curves, this was less true for IgA, particularly the anti-RBD IgA response. This is because some of our negative controls, irrespective of whether they were collected during the pandemic (unexposed negatives) or prior to the pandemic, exhibit levels of anti-RBD IgA that approach 50% of the pooled control saliva (see Fig. 4H). It is unclear why this would occur for only the IgA/RBD combination. Presumably these are cross-reactive IgA that bind to SARS-CoV-2 RBD. Of interest, thus far SARS-CoV-2 neutralizing antibodies appear to have limited somatic hypermutation (*36*, *37*), suggesting that they may originate from a naïve repertoire or from B cells that have been activated in extrafollicular responses where somatic hypermutation is limited. It is tempting to speculate that these pre-existing IgA antibodies may provide some stop-gap protection against SARS-CoV-2 in the oral cavity, and if so, it is essential to ascertain their original antigenic specificity. Future work is required to confirm these results in a greater array of subjects and using different sources of RBD antigen.

Our findings that the IgG response to SARS-CoV-2 antigens is stable over a 3-month period are consistent with other studies who likewise noted durability in the IgG response to the spike trimer (*8*–*13*). These data and ours contrast with those of Long *et al*., who showed rapid decay of antibody levels when profiling the response to a linear peptide motif of the C-terminal part of the spike protein (*14*) instead of the spike trimer used here, and it is possible that the antigen selection accounts for some of the differences. However, this does not explain discrepant results with respect to the anti-NP response in the serum, which we find also largely persisted over the 3-month period. One potential difference that could explain these divergent results is that we employed a sensitive and robust chemiluminescence plate-based ELISA whereas Long *et al*. employed magnetic chemiluminescence enzyme immunoassay kits with immobilized recombinant or peptide antigens.

A limitation of our study is that we have not looked beyond the day 115 PSO – our collections began in mid-March 2020 – and it is entirely plausible that antigen-specific IgG levels will eventually wane with time. Although IgG antibodies to spike remained fairly stable, even at day 115 PSO, our surrogate neutralization assay revealed a dip in activity in the last time interval bin (days 116 - 115 PSO), consistent with some previous studies (*9*, *13*, *14*). This final collection interval is not as well powered as the other bins, thus this requires further investigation. Nevertheless, a dip in neutralization activity using the surrogate assay does mirror the significant reduction in antigen-specific IgA (and IgM). The contributions of these isotypes to the overall neutralization activity at different time points after infection remains to be assessed. Indeed, IgA is an important mediator of protection against gastrointestinal viruses (*38*), is essential in achieving immunity against avian viruses (*39*), has been shown to contribute to the neutralizing antibody (nAb) response to SARS-CoV-2 (*28*), and may even be a more potent nAb isotype than IgG (*40*). In addition, a monoclonal antibody cloned from B cells derived from SARS-CoV-infected humanized mice was found to provide cross-reactive neutralizing activity to SARS-CoV-2 when engineered on the IgA backbone, and this neutralizing activity was further enhanced if the IgA was co-expressed with J chain to produce dimeric IgA and secretory component to produce secretory IgA – the form of IgA that is secreted at mucosal surfaces (*41*). Although Sterlin *et al*. show that the initial IgA plasmablast response quickly declines, IgA-producing plasma cells have been shown to persist for decades in the gut mucosae of humans (*42*), and these will not be readily measurable in the blood. Indeed, we found that of all 3 isotypes measured, antigen-specific IgA levels in the saliva exhibited the poorest correlation with antigen-specific IgA levels in the serum. When combined with the parallel formation of re-activatable memory B cells (*43*), many of which will be tissue-resident (*25*), the host has excellent mechanisms for mounting swift and robust humoral immunity upon pathogen re-exposure that may be missed using blood-based measurements. An epidemiological study that prospectively follows confirmed COVID-19 cases for several months will determine if these immunological principles hold true in the context of SARS-CoV-2 infection.

In conclusion, our study provides evidence that the IgG response to SARS-CoV-2 spike persists in the saliva and the serum, and that this response can be correlated between the two biofluids, particularly for IgG. Given that the virus can also be measured in saliva by PCR (*16*–*19*), using saliva as a biofluid for both virus and antibody measurements may have some diagnostic value. Since SARS-CoV-2 initially replicates in the oro- and nasopharyngeal tracts, in the future it will be critical to characterize the nature and kinetics of salivary antibodies at the earliest time points post-infection in contact-traced individuals in order to determine if there are correlates of protection that impact viral setpoint and COVID-19 disease progression.

## MATERIALS AND METHODS

### Study Design

This observational study focused on monitoring the levels of antibodies to SARS-CoV-2 antigens in serum and saliva of patients with confirmed SARS-CoV-2 infection. At the onset of the study, we set to determine: 1) what are the kinetics of antibody production and decline in saliva and serum specimens from patients with COVID-19 during the first 3+ months of infection; 2) whether these levels are affected by disease severity, sex, or age; 3) whether saliva can be used as an alternative biofluid for monitoring the immune response in patients with COVID-19. Assay development was performed for each individual ELISA by assessing the classification of positives and negative samples (see definition below for serum and saliva assays) at each of the observed colorimetric or chemiluminescent values, and setting a threshold (1% for serum and 2% for saliva, respectively) for definition of positives. Irrespective of this positive/negative definition, all values are reported. The protein-based surrogate neutralization ELISA (snELISA) development and benchmarking against viral neutralization assay was described previously (*24*). Samples for profiling were recruited through the Toronto Invasive Bacterial Diseases Network in metropolitan Toronto; all samples for which a PCR positive result and for which the biofluid (serum or saliva) was available were included. Data was analyzed without exclusion of outliers to avoid biasing the study. For the saliva and snELISA assays, each sample was analyzed once, through a multipoint dilution curve; for the serum-based ELISA, a single-point ELISA was performed in duplicates, and the results averaged. No randomization was performed, since this is an observational study.

### Recruitment and participants – COVID19 patients

Acute and convalescent serum and saliva samples were obtained from patients identified by surveillance of COVID-19 (confirmed by PCR; in- and out-patients) by the Toronto Invasive Bacterial Diseases Network in metropolitan Toronto and the regional municipality of Peel in south-central Ontario, Canada (REB studies #20-044 Unity Health Network, #02-0118-U/05-0016-C, Mount Sinai Hospital). Consecutive consenting patients admitted to four TIBDN hospitals were enrolled: these patients had serum and saliva collected at hospital admission, and survivors were asked to submit repeat samples at 4-12 weeks PSO. Consecutive out-patients diagnosed at the same 4 hospitals prior to March 15^th^ and on a convenience sample of later days were approached for consent to collect serum and saliva at 4-12 weeks PSO. Patients were interviewed and patient charts reviewed to determine age, sex, symptom onset date, and disease severity (mild, moderate, and severe). For this study, disease was considered mild if it did not require hospitalization, moderate if it required hospitalization but not intensive care unit (ICU) admission, and severe if it required ICU care. Specimens were considered acute if they were collected less than 21 days PSO, and convalescent if they were collected 21 or more days PSO. From March 10-April 14, patients were asked to provide a 5 ml sample of saliva in a sterile specimen container, and 2.5 mls of phosphate buffered saline was added to reduce viscosity for PCR testing. From April 16^th^ on, saliva specimens were collected in Salivette® tubes (Sarstedt, Numbrecht, Germany). All specimens were aliquoted and stored frozen at -80°C prior to analysis.

Additional positive samples for test development were obtained through the Canadian Blood Services. Specimen-only serum donations were collected from individuals with a self-declared SARS-CoV-2-positive nucleic acid test. Collections occurred two weeks or more after cessation of clinical symptoms.

### Recruitment and participants – control saliva and serum

Control saliva samples were collected from unexposed, asymptomatic individuals residing in an area of very low COVID-19 case numbers (Grey County, Ontario) and throughout the Greater Toronto Area (GTA) (REB study# 23901 University of Toronto).

Control serum samples were from patients enrolled in cancer or birth cohort studies prior to COVID-19 (prior to November 2019; REB studies #01-0138-U and #01-0347-U, Mount Sinai Hospital) and archived frozen in the LTRI Biobank, or from previous studies of the immune system or systemic lupus acquired prior to November 2019 (REB studies #31593 University of Toronto, #05-0869, University Health Network).

### Study Approval

All samples were collected after Research Ethics Board (REB) review (see Sample section above for the individual REB approval numbers). The serum ELISA assays were performed at the Lunenfeld-Tanenbaum Research Institute with Mount Sinai Hospital (MSH; Toronto, ON) Research Ethics Board (REB) approval (study number: 20-0078-E). External samples were transferred through Material Transfer Agreements as appropriate. All research has been performed in accordance with relevant guidelines and regulations. All participants have provided informed consent. The samples were de-identified prior to transfer to the assay laboratory.

### Sample collection, handling and viral inactivation – serum

Serum (and in some cases plasma) was collected using standard procedures at the collection sites and transferred to the testing lab on dry ice. Inactivation of potential infectious viruses in plasma or serum was performed by incubation with Triton X-100 to a final concentration of 1% for 1 hour prior to use (*44*).

### Antigen production – serum assays

Spike trimer was expressed as follows: the SARS-CoV-2 spike sequence (aa 1-1208 from GenBank accession number MN908947 with the S1/S2 furin site (residues 682–685) mutated [RRAR->GGAS] and K986P / V987P stabilizing mutations was codon-optimized (*Cricetulus griseus* codon bias) and synthesized by GenScript. To stabilize the spike protein in a trimeric form, the cDNA was cloned in-frame with the human resistin cDNA (aa 23-108) containing a C-terminal FLAG-(His)_6_ tag (*Cricetulus griseus* codon bias, GenScript) into a modified cumate-inducible pTT241 expression plasmid and transfected in CHO^2353^ cells followed by methionine sulfoximine selection for 14 days to generate a stable CHO pool. This CHO pool allows for cumate-inducible trimeric spike expression from the CR5 promoter as described in Poulain *et al*. (*45*–*47*). Cell culture was harvested 8-10 days post-cumate induction and secreted spike trimer present in the clarified medium purified by immobilized metal-affinity chromatography (Ni-Excel resin; Cytiva). Purified trimeric spike was buffer exchanged in PBS and store as aliquots at -80°C. The purified spike protein integrity and purity was analyzed by SDS-PAGE and analytical size-exclusion ultra-high performance liquid chromatography (SEC-UPLC). The SEC-UPLC was run in PBS + 0.02% Tween-20 on an 4.6 × 300 mm Acquity BEH450 column (2.5 μm beads size; Waters Limited, Mississauga, ON) coupled to a MALS detector (miniDAWN^TM^) and an Optilab® T-rEX^TM^ refractometer (both from Wyatt Technology) and the spike trimer eluted as a major (>95% integrated area) symmetrical peak of 490 kDa with less than 3% aggregates (not shown). RBD was expressed as for the saliva assay, but left non-biotinylated, as in (*24*).

Nucleocapsid (aa 1-419 from the pEntry-N (closed) Open Reading Frame (a kind gift from Dr. Frederick P. Roth (*48*)) was cloned into pDEST585 gift of Jim Hartley, internal ID V2097) as a HIS-GST-TEV fusion using LR-clonase. The resulting expression vector was confirmed by restriction digest, expressed in *E. coli* BL21(DE3) Codon+ cells (Agilent Technologies) and induced with 0.25 mM isopropyl 1-thio-β-D-galactopyranoside (IPTG) for 16 hours at 18°C. Harvested cells were resuspended in 20 mM HEPES pH 7.5, 400 mM NaCl, 5 mM imidazole and lysed by passage through a cell homogenizer (Avestin Inc.). Following centrifugation at 30,000 g, supernatant was passed through a 0.45 μM PVDF filter and applied to a HiTrap nickel chelating HP column (GE Healthcare). Protein eluted with buffer containing 300 mM imidazole was incubated overnight with Tobacco Etch Virus (TEV) protease. Following cleavage of the His-Tag, protein was dialyzed in 20 mM HEPES pH 7.5, 50 mM NaCl and flowed over a 5 ml HiTrap nickel chelating column to remove His-GST. Nucleocapsid protein was further purified by ion exchange on a mono-S column (GE Healthcare) equilibrated in 20 mM HEPES pH 7.5, 50 mM NaCl, 1 mM DTT and eluted with a gradient to 500 mM NaCl. Purified Nucleocapsid protein was concentrated to 6 mg/mL and stored at -80°C.

### Enzyme-linked immunosorbent assays for detecting antigen-specific IgG and IgA in serum or plasma

A manual colorimetric ELISA assay (similar to (*3*)) was first implemented in 96-well plates using the RBD and spike non-biotinylated antigens described here for the detection of IgG (also see (*24*)). Briefly, concentrations and incubation times were optimized to maximize the separation between anti-RBD or anti-spike trimer levels in convalescent plasma or serum from that of pre-COVID era banked serum while maintaining the required levels of antigens as low as possible. 75 ng and 200 ng of RBD and spike, respectively, were first adsorbed onto 96-well clear Immulon 4 HBX (Thermo Scientific, #3855) plates in PBS overnight at 4°C, then washed three times with 200 μl PBS+ 0.1% Tween-20 (PBS-T; Sigma). Plates were blocked with 3% w/v milk powder (BioShop Canada Inc., #ALB005.250, lot #9H61718) in PBS for 1–2 hours and washed three times with 200 μl PBS-T. Patient samples (pre-treated with 1% final Triton X-100 for viral inactivation) diluted 1:50 in PBS-T containing 1% w/v milk powder were then added to the plates and incubated for 2 hours at room temperature (50 μl total volume): technical duplicates were performed unless otherwise indicated. Positive and negative control recombinant antibodies and serum samples were added to each plate to enable cross-plate comparisons. Wells were washed three times with 200 μl PBS-T. Goat anti-human anti-IgG (Goat anti-human IgG Fcγ -HRP, Jackson ImmunoResearch, #109-035-098) at a 1:60,000 dilution (0.67 ng/well) in 1% w/v milk powder in PBS-T was added and incubated for 1 hour. Wells were washed three times with 200 μl PBS-T, and 50 μl of 1-StepUltra TMB-ELISA Substrate Solution (ThermoFisher, #34029) was added for 15 min at room temperature and the reaction was quenched with 50 μL stop solution containing 0.16N sulfuric acid (ThermoFisher, #N600). The plates were read in a spectrophotometer (BioTek Instruments Inc., Cytation 3) at 450 nm. For all ELISA-based assays, raw OD or luminescence values had blank values subtracted prior to analysis. All data were normalized to the positive serum control pool (single point) on each plate and expressed as a ratio to this control (ratio-converted ELISA reads). The assay performance was assessed by precision-recall analysis of ratio-expressed values (Figure S1, S3).

The assay was then re-designed to be conducted in a customized robotic platform using a 384-well plate format, first by simply scaling down the volume/amounts used, and then switching to a chemiluminescent substrate for detection, and re-optimizing the amounts per well of antigens and secondary antibodies’ dilutions to use. A chemiluminescent substrate is ideally-suited for automated ELISAs, because it offers a higher sensitivity and a better dynamic range than standard colorimetric assays. Furthermore, the reaction does not need to be stopped (e.g., with robotics-incompatible acids) and the luminescence signal is stable for at least 60 min. For all steps, liquid dispensers (Beckman Biomek NXp or ThermoFisher Multidrop Combi) and washer (Biotek 405 TS/LS LHC2) were used on a F7 robotic platform available at the Network Biology Collaborative Centre (nbcc.lunenfeld.ca). Each step of the methods to evaluate the different antigen and antibody class combinations was optimized and routine quality control tests were performed for all dispensing steps.

For automated ELISAs, LUMITRAC 600 high-binding white polystyrene 384-well microplates (Greiner Bio-One, through VWR #82051-268) were pre-coated overnight with 10 μl/well of RBD (25 ng) or spike (50 ng). The next day, the wells were washed 4 times (a BioTek washer is used for all washing steps, and all washes are performed with 100 μl PBST). Wells were blocked for 1 hour at room temperature in 80 μl 5% Blocker^TM^ BLOTTO (Thermo Scientific, #37530), then washed 4 times. 10 μl Triton X-100 inactivated serum (or plasma) samples diluted 1:40 in 1% BLOTTO in PBS-T were added to each well from 96-well sample source plates and incubated for 2 hours at room temperature. Positive and negative controls used on each plate are described below. After washing 4 times, 10 μl of one of the following secondary antibodies (all from Jackson ImmunoResearch) diluted in 1% BLOTTO in PBS-T were added at the indicated concentrations followed by incubation for 2 hours at room temperature: Goat anti-human IgG Fcγ – HRP (#109-035-098; 1:40,000 or 0.2 ng per well), Goat anti-human IgM Fcμ – HRP (#109-035-129; 1:12,000 or 0.66 ng per well) or Goat anti-human IgA α chain - HRP (#109-035-127; 1:10,000 or 0.8 ng per well). After 4 washes, 10 μl of SuperSignal ELISA pico Chemiluminescent substrate (diluted 1:4 in water) was added, followed by a short mix for 10s at 900 rpm, and incubation at room temperature for 5 min. Luminescence was read on an EnVision (Perkin Elmer) plate reader at 100 ms/well using an ultra-sensitive luminescence detector. All automated assays were performed in technical duplicates, processed on different days. Blank values were subtracted for all raw reads prior to data analysis, and the values were expressed as a ratio of the positive reference serum pool on the same plate (see below).

Quality controls and normalization of the samples in the automated assays were as follows: a standard curve with recombinant antibodies reacting to spike RBD or spike S1 was included on each plate. Antibodies used for the standard curves were: Human anti-spike S1 IgG (A02038, GenScript), anti-spike S1 IgM (A02046, GenScript) and Ab01680 anti-spike IgA (Ab01680-16, Absolute Antibody), all used at 0.5 to 10ng per well. Negative antibody controls were immunoglobulins from human serum (I4506 human IgG, I8260 human IgM, and I4036 human IgA, from Millipore-Sigma). A positive and negative control pool of 4 patient samples each was created and added in each plate at a single point concentration for normalization, or as a standard curve, starting with a 1:40 dilution. For all assays, a standard curve is generated by first plotting the mean of the blank-subtracted recombinant antibodies, plotted against antibody amounts (in ng) (or the positive pooled sera), and the linearity of the curve and comparison to previous runs is assessed, alongside the confirmation that the positive and negative pool sample fall within the expected range of the standard curve [%CV should be 10-15% or less]. Figure S8 displays the dilution curves for the assays shown here and Figure S9 represents the ratio distributions across the samples used here. See Table S4 for all data.

### Surrogate neutralization enzyme-linked immunosorbent assays (snELISA)

To provide a measurement of the potential of the serum antibody to neutralize SARS-CoV-2, we employed a surrogate neutralization ELISA (snELISA) we described recently (*24*). Briefly, 20 samples per PSO time bin were randomly selected and subjected to a four-point snELISA assay (starting with 4 μl serum) evaluating the capacity of serum antibodies to prevent the association of biotinylated ACE2 to immobilized RBD. Areas under the curves of the last two points were tabulated as an “integrated score” value: lower values correspond to a stronger displacement.

### Sample collection and handling - saliva study

With the exception of some samples that were acquired early on in the pandemic (cohort 1), Salivette® tubes were used to collect samples according to manufacturer instructions (Sarstedt, Montreal, Quebec). These tubes include a cotton swab that participants are instructed to chew for set amount of time. The swab is then transferred into an inner tube which is then inserted into an outer tube that catches liquid saliva upon centrifugation at 1000 × g for 3 min (Centrifuge 5910 R, Eppendorf). Salivary flow was controlled by establishing a fixed amount of collection time (2 min) for each subject as previously recommended (*24*, *49*). For the early pandemic subjects that were not given Salivettes® and used in our pilot study (cohort 1), these subjects expectorated directly into a 15 mL conical tube containing 2.5 mL of phosphate-buffered saline (PBS). Prior to saliva collection, healthy subjects confirmed they had fasted, refrained from taking oral medication, and had not brushed their teeth for a minimum of 30 min.

### Viral inactivation in saliva samples

Following centrifugation, all saliva samples, regardless of their SARS-CoV-2 PCR status, underwent viral inactivation by treating with Triton^®^ X-100 (BioShop, CAT# TRX506.100). 10% Triton X-100 (diluted 1:10 from stock) was added to all samples to a final dilution of 1% Triton X-100 and incubated for 1 hour at room temperature. Inactivated samples were immediately frozen and stored at -80°C. Heat inactivation for 30 min at 65°C was found to destroy the IgG and IgA signal against RBD and was therefore not used (Figure S5). The efficiency of virus inactivation in a saliva medium is shown in Table S3. Specifically, we assessed the treatment of saliva collected from healthy individuals using two different methods (Salivette® vs. direct saliva collection into a tube). These samples were spiked with known amounts of SARS-CoV-2 viral stock and then treated with 1% Triton X-100 for 30 min, 1 hour or 2 hours. Vero-E6 cells (ATCC_®_ CRL-1586^TM^) were used to determine outgrowth of virus. Cells were maintained in Dulbecco’s Modified Eagle’s Media (DMEM) supplemented with L-glutamine, penicillin/streptomycin and 10% fetal bovine serum (FBS). SARS-CoV-2 virus (isolate SB3) was isolated in-house (*50*). Briefly, viral stocks were created after isolation of virus from a clinical sample in Toronto, Ontario, Canada. Viral stock was expanded using Vero E6 as previously described such that stored aliquots of stock contain 2% FBS. Initial experiments were done with Triton X-100 (Sigma-Aldrich) serially diluted and applied to Vero-E6 cells in 96-well flat bottom plates to determine the minimum concentration required to prevent toxicity to cells. Furthermore, we have also determined if neat saliva itself could be cytotoxic to Vero-E6 cells by providing healthy donor saliva alone or treated with Triton X-100 ranging from final Triton-X100 concentration of 0.03%, 0.01%, 0.001% and 0.0001% (v/v). Since initial Triton X-100 experiments showed that toxicity is averted at 0.03% (v/v), we proceeded to use this concentration as the point of dilution to prevent any Triton X-100 mediated toxicity.

### Antigen production – saliva assay

The expression, purification and biotinylation of the SARS-CoV-2 RBD and spike ectodomain were performed as recently described (*24*, *49*). The human codon optimized cDNA of the SARS-CoV-2 spike protein was purchased from Genscript (MC_0101081). The soluble RBD (residues 328-528, RFPN...CGPK) was expressed as a fusion protein containing a C-terminal 6xHis tag followed by an AviTag. The soluble trimeric spike protein ectodomain (residues 1-1211, MFVF...QYIK) was expressed with a C-terminal phage foldon trimerization motif followed by a 6xHis tag and an AviTag. To help stabilize the spike trimer in its prefusion conformation, residues 682–685 (RRAR) were mutated to SSAS to remove the furin cleavage site and residues 986 and 987 (KV) were each mutated to a proline residue (*51*). Stably transfected FreeStyle 293-F cells secreting the RBD and soluble spike trimer were generated using a previously reported piggyBac transposon-based mammalian cell expression system (*52*). Protein production was scaled up in 1L shake flasks containing 300 mL FreeStyle 293 medium. At a cell density of 10^6^ cells/mL, 1 μg/mL doxycycline and 1 μg/mL Aprotinin were added. Every other day 150 mL of medium was removed and replaced by fresh medium. The collected medium was centrifuged at 10000 × g to remove the cells and debris and the His-tagged proteins were purified by Ni-NTA chromatography. The eluted protein was stored in PBS containing 300 mM imidazole, 0.1% (v/v) protease inhibitor cocktail (Sigma, P-8849) and 40% glycerol at -12°C. Shortly before use, the RBD and spike proteins were further purified by size-exclusion chromatography on a Superdex 200 Increase (GE healthcare) or Superose 6 Increase (GE healthcare) column, respectively. Purity was confirmed by SDS-PAGE. For the spike protein, negative stain electron microscopy was used show evidence of high-quality trimers. The Avi-tagged proteins, at a concentration of 100 μM or less, were biotinylated in reaction mixtures containing 200 μM biotin, 500 μM ATP, 500 μM MgCl_2_, 30 μg/mL BirA, 0.1% (v/v) protease inhibitor cocktail. The mixture was incubated at 30°C for 2 hours followed by size-exclusion chromatography to remove unreacted biotin.

### Enzyme-linked immunosorbent assays for detecting total IgA, IgG and IgM in saliva

Quantitative total IgA, IgG, and IgM analyses were performed on the same samples used for detection of anti-RBD and anti-spike Ig described below. Anti-human Ig antibody (Southern Biotech, 2010-01) diluted 1:1000 in PBS was added to 96-well Nunc MaxiSorp plates (ThermoFisher, 44-2404-21). PBS alone was added to control wells. Plates coated overnight at 4°C. Following coating, plates were blocked using 200 μl/well of 5% BLOTTO for 2 hours at 37°C. Samples were diluted in 2.5% BLOTTO, the volume of which was variable depending on the dilutions being tested per sample, per antibody isotype. Standards (purified IgA, IgG and IgM purchased from Sigma-Millipore: IgA, I4036, IgG, I2511, and IgM, I8260) were prepared in 2.5% BLOTTO ranging from 100 ng/mL to as low as 0.78 ng/mL depending on the antibody isotype being run. Upon discarding the blocking solution from the plate, 50 μl of diluted samples and 50 μl of each standard concentration solution were immediately transferred to wells and incubated for 2 hours at 37°C. Following incubation, wells were washed with 200 μL of PBS-T. HRP-conjugated secondary antibodies against IgA, IgG, and IgM (goat anti-human IgA- and IgG-HRP, Southern Biotech, IgA: 2053-05, IgG: 2044-05, IgM: 2023-05) were added to the appropriate wells at 1:1000 in 2.5% BLOTTO and incubated for 1 hour at 37°C. Development of the plate was done by adding 50 μL of 3,3′,5,5′-tetramethylbenzidine (TMB) Substrate Solution (ThermoFisher, 00-4021-56) onto plates. Reaction vas then stopped by adding 50ul/well of 1N H_2_SO_4_ Optical density (OD) was read at a wavelength of 450 nm on a spectrophotometer (OD_450_). A four-parameter logistic curve was used to determine the line of best fit for the standard curve, and sample Ig quantities were interpolated accordingly to determine final concentrations in μg/ml. The few samples from patient or control groups that exhibited quality control issues (extremely low to negative IgA levels) were excluded from further analysis.

### Enzyme-linked immunosorbent assay for detecting albumin in saliva

Salivary albumin was measured for Cohort 1 using a purchased Human Albumin ELISA Kit (Abcam, ab108788). Assay was performed according to manufacturer instructions included with the kit.

### Enzyme-linked immunosorbent assays for detecting antigen-specific IgG, IgA and IgM in saliva

96-well plates pre-coated with streptavidin (ThermoFisher, 436014) were used for all assays. Without the biotin-streptavidin system, the anti-S/RBD IgG, IgA, and IgM signals obtained from COVID-19 patient saliva were undetectable. Based on titrations of antigens using saliva from convalescent COVID-19 patients, 2 μg/ml biotinylated-RBD and 20 μg/ml biotinylated-spike protein solutions were prepared in sterile PBS one day prior to starting the assay. From these dilutions, 50 μl of each were added to appropriate wells in our plates, resulting in 100 ng of biotinylated-RBD and 1 μg of biotinylated S proteins applied to the appropriate wells (see Figure S5 for RBD titration, spike titration not shown). Control wells of sterile PBS rather than biotinylated antigen were reserved for each patient and control sample. A few wells with the biotinylated antigen but with no sample added were reserved as negative internal controls for the reagents on the assay. Plates were incubated overnight at 4°C to allow sufficient coating of the antigen. 200 μL of 5% BLOTTO (5% w/vol skim milk powder (BioShop, CAT# SKI400.500) in sterile PBS) was subsequently added to each well to prevent nonspecific interactions, followed by a 2-hour incubation at 37°C. Blocking solution was discarded immediately from plates prior to addition of samples to wells. Newly thawed saliva samples were centrifuged at 8000 rpm for 4 min (Microcentrifuge 5418, Eppendorf), and appropriately diluted using 2.5% BLOTTO at dilutions ranging from 1:2 to 1:20 depending on the cohort being tested and the way in which saliva was collected. To reduce anti-streptavidin reactivity in the saliva, diluted samples were applied to streptavidin-coated plates with no antigen and allowed to incubate for 30 min at 37°C. Subsequently 50 μL of samples were transferred from the pre-adsorption plate into antigen-coated plates and incubated for 2 hours at 37°C. PBS+0.05% Tween 20 (BioShop, CAT# TWN510) (PBS-T) was used for washing plates between steps. Horseradish peroxidase (HRP)-conjugated goat anti human-IgG, IgA, and anti-IgM secondary antibodies (Southern Biotech, IgG: 2044-05, IgA: 2053-05, IgM: 2023-05) were added to wells at dilutions of 1:1000, 1:2000 and 1:1000 in 2.5% BLOTTO, respectively, and incubated for 1 hour at 37°C. Development of the plates was performed as described in the section above.

For cohort 1, because some samples had been collected in cups and were therefore diluted, normalization to a separate variable was performed. The resulting OD from antigen-specific IgA and IgG was subtracted from the OD for the PBS control wells for each sample and subsequently normalized to albumin levels or total IgA and IgG levels, respectively (see below). IgM was not calculated for cohort 1 due to lack of remaining sample from the COVID-19 patients. For cohort 1, raw OD_450_ measurements obtained from PBS-coated wells corresponding to each sample diluted at 1:5 (“background signal”) was subtracted from readings obtained from antigen-coated wells at each of two dilutions (1:5, 1:10). The OD from the highest concentrated saliva dilution (1:5 for samples collected by Salivette®, or 1:2 for saliva samples collected in cups and prediluted) was normalized to the total IgG, total IgA, or albumin content in each saliva sample. A small number of samples (n=9 from negative controls and n=4 from patients) exhibited high OD values that did not titrate and coincided with high OD levels when plated without antigen (PBS control). These were excluded from the analysis.

For cohort 2, raw OD_450_ measurements obtained from PBS-coated wells corresponding to each sample diluted at 1/5 (“background signal”) was subtracted from readings obtained from antigen-coated wells at each of three dilutions (1:5, 1:10, 1:20). For each plate, a sample of pooled saliva from COVID-19 acute and convalescent patients was likewise plated at 1/5 with no antigen (PBS control), as well as with antigens at 1:5, 1:10 and 1:20. The area under the curve was calculated based on the background subtracted values from all three dilutions for each sample. A pooled sample of positive control saliva was run on each plate and analyzed in the same manner. Each sample within a given plate was then normalized to the pooled positive control saliva for that particular plate and expressed as a percentage. For simplicity, we denoted this percentage as an “integrated score” (Figure S6A). By using the same positive control that we ran in every single plate, we determined that intra-assay precision was always greater than 90% between plates. Reproducibility between plates was determined by a coefficient of variation of less than 10% through all the plates. A small number of samples (n=6 from negative controls and n=2 from patients) exhibited high OD values that did not titrate and coincided with high OD levels when plated without antigen (PBS control) (Figure S6B). These were excluded from the final analysis.

### Receiver-Operating Characteristic (ROC) curves

For serum and plasma sample analysis, samples acquired prior to November 2019 (pre-COVID) were labeled true negatives while convalescent samples from patients with PCR-confirmed COVID-19 were labeled true positives. For saliva samples, all samples from patients with PCR-confirmed COVID-19 collected more than 10 days PSO were considered true positives, and saliva collected before 2020 and from unexposed, asymptomatic individuals in March of 2020 were labeled true negative for ROC analysis. Ratio-converted ELISA reads (colorimetric or chemiluminescent) were used for ROC analysis in the easyROC webtool (v 1.3.1) with default parameters (https://journal.r-project.org/archive/2016/RJ-2016-042/index.html). Non-parametric curve fitting was applied alongside DeLong’s method for standard error estimation and confidence interval generation.

### Statistical analysis

For total IgA, IgG and IgM readouts in saliva, raw OD_450_ measurements obtained from PBS-coated wells (“background signal”) was subtracted from readings obtained from anti-human Ig-coated wells with saliva samples added (OD_450_ of sample – OD_450_ of PBS-coated well). Total IgA, IgG and IgM quantifications were determined relative to standard wells present on each plate. A four-parameter logistic curve was used to determine the line of best fit for the total IgA/M/G standard curves, and sample Ig quantities were interpolated accordingly, using Prism (GraphPad), Version 8.3.

For the analysis in the antigen ELISA of cohort 1, the raw OD_450_ measurements from the PBS-coated wells with sample added at 1/5 dilution (“background signal”) was subtracted from each of the saliva sample dilutions (1:5, 1:10) added to wells coated with protein (OD_450_ of sample from coated well – OD_450_ of PBS coated well with sample). The blank-corrected OD from the 1:5 sample dilution in the antigen-specific IgA and IgG were subsequently normalized to the concentration of total IgA and IgG, respectively, for cohort 1. An additional normalization strategy consisted of normalizing the blank corrected OD from antigen-specific IgA and IgG to the concentration of albumin for cohort 1.

For the analysis in the antigen ELISA of cohort 2, the raw OD_450_ measurements from the PBS-coated wells with sample added at 1:5 dilution (“background signal”) was subtracted from each of the saliva sample dilutions (1:5, 1:10, 1:20) added to wells coated with protein (OD_450_ of sample from coated well – OD_450_ of PBS coated well with sample). For cohort 2, the blank corrected OD antigen-specific IgA, IgG, and IgM OD values across three dilutions were used to calculate the integrated score for each sample. The sample area under the curve was normalized to the score of a positive pool of saliva samples used as an internal standard across all plates. The values were expressed as a percentage and denoted as an integrated score.

For serum, raw OD_450_ measurements for IgG, IgA and IgM on spike, RBD and NP from either the manual or automated platforms were subtracted from wells coated with PBS. A pool of serum samples that previously exhibited high levels of IgGs to all antigens was used as an internal standard across all plates, and a relative ratio between blank-adjusted measurements (OD_450_ or chemiluminescent reads) of patient samples and measurements of this positive pooled standard are reported as “ratio-converted ELISA reads.” Serum data were analyzed in R using version 4.0.1. Median antibody levels between negative and positive subject groups (saliva) or negative, acute and convalescent subject groups (blood) were compared using Mann Whitney U tests. These analyses were performed in Prism (GraphPad), Version 8.3.

The relationship between time PSO and antibody levels in the convalescent period was examined in multivariable linear regression models that adjusted for age, sex, and disease severity. For serum samples, seven multivariable linear regression models were constructed (one for each of anti-RBD IgA, anti-S IgA, anti-RBD IgG, anti-S IgG, anti-RBD IgM, anti-spike IgM, neutralizing antibody). Generalized estimating equations were used (proc genmod in SAS with exchangeable correlation matrices) to account for patient-level clustering. Antibody levels were transformed as appropriate to achieve heteroscedasticity, and the variance inflation factors for all covariates confirmed to be <5 to verify absence of multicollinearity. For saliva samples, six multivariable linear regression models were similarly constructed; however, only the first convalescent sample for each patient was included in the analysis (proc glm in SAS).

## Supplementary Materials

immunology.sciencemag.org/cgi/content/full/5/52/eabe5511/DC1 Fig. S1. Development and validation of manual colorimetric and automated chemiluminescent assays for monitoring RBD and spike trimers antibodies in serum or plasma. Fig. S2. Correlations between antibody levels and day of symptom onset to sample collection. Fig. S3. IgG and IgA responses to the Nucleocapsid antigen of SARS-CoV-2 in serum. Fig. S4. Effect of heat versus detergent inactivation of saliva samples on the detection of anti-RBD antibodies in a manual, colorimetric ELISA. Fig. S5. IgG and IgA levels against SARS-CoV-2 antigens in the saliva of cohort 1. Fig. S6. Strategy for calculating an integrated score in antigen-specific saliva assay. Fig. S7. Validation of manual colorimetric assays for monitoring RBD and spike trimers antibodies in saliva. Fig. S8. Dilution curves of positive control ELISAs for serum assays. Fig. S9. Distribution of the intensity values across serum assays for negative controls and samples from patients with COVID-19 infection. Table S1. ROC statistics table for ELISAs conducted on serum/plasma derived samples. Table S2. Testing effect of Triton-X treatment on saliva samples. Table S3. ROC statistics table for ELISAs conducted on saliva samples. Table S4. Raw data file (Excel spreadsheet).
